# Bioengineered human blood vessels to treat hospital-acquired vascular complications

**DOI:** 10.1016/j.jvscit.2025.101976

**Published:** 2025-09-08

**Authors:** YingWei Lum, Ernest E. Moore, Rishi Kundi, Jonathan Morrison, Jamie T. Shores, Laura E. Niklason, Shamik Parikh

**Affiliations:** a Division of Vascular Surgery and Endovascular Therapy, Johns Hopkins Bayview Medical Center/Johns Hopkins Hospital, Baltimore, MD; b Department of Surgery, Ernest E. Moore Shock Trauma Center at Denver Health, Denver, CO; c Division of Vascular and Endovascular Trauma surgery, R. Adams Cowley Shock Trauma Center, Baltimore, MD; d Microvascular Surgery, The Orthopedic Center of St Louis, St Louis, MO; e Humacyte Global, Inc, Durham, NC

**Keywords:** Vascular graft, Iatrogenic injury, Vascular surgery complication, Bioengineered blood vessel, Engineered artery, Tissue engineering

## Abstract

**Background:**

Complications of vascular surgery and vascular procedures, including iatrogenic injuries, planned oncological tumor resections, and steal syndrome after arteriovenous access placement, are increasingly common in modern medical care. Harvesting of autologous vein to address these consequences and complications produces injury to the patient, and suitable vein may not be accessible in the urgent/emergent setting.

**Methods:**

The objective is to evaluate the performance of the acellular tissue engineered vessel (ATEV) in the repair of sequelae and complications of vascular procedures. CLN-PRO-V005 (clinicaltrials.gov NCT03005418; “V005”) is a multicenter, single-arm clinical trial at 19 level I trauma centers in the United States and Israel, that evaluated the safety and efficacy of the ATEV in the repair of arterial injuries. Twelve patients analyzed for this report sustained hospital-acquired iatrogenic injury, or incurred sequelae or complications of vascular surgical procedures, and had no autologous vein for repair. Results from these 12 patients have not been previously reported. Patients received the ATEV, which is a bioengineered vascular conduit grown from human vascular cells. Key outcome measures were patency, limb salvage, conduit infection, and patient survival, at day 30 and at completion of follow-up. Patients were followed for 36 months, or until withdrawal, death. or formal data closure.

**Results:**

The 12 patients in the V005 study with hospital-acquired iatrogenic injuries or consequences of vascular procedures were 50% male, 58% Caucasian, and aged 57.0 ± 16.9 years. Of all cases, 67% occurred in the lower extremities. The mean length of ATEV implanted was 12.1 ± 9.8 cm. The average follow-up was 23.3 ± 15.9 months. At day 30, all 10 evaluable patients had documented patency; 1 patient withdrew consent at day 2 with a patent conduit, and 1 patient died on day 12 with a patent conduit. At the conclusion of follow-up or at data cutoff, death, or withdrawal, 11 of 12 total patients retained patency, of whom 7 had primary patency, 3 had primary-assisted patency, 1 had secondary patency, and 1 patient underwent explantation of the ATEV. There were no losses of the treated limbs. There were no confirmed infections of the ATEV conduit reported for any patient. Four of the 12 patients died during follow-up, with no deaths related to ATEV.

**Conclusions:**

ATEV can provide limb salvage and durable patency in patients experiencing iatrogenic injury or complications and sequelae of planned vascular procedures.

**Article Highlights**

• **Type of Research:** Multicenter subgroup analysis of prospective, single-arm trial
• **Key Findings:** Complications and sequelae of vascular procedures in the extremities in 12 patients were treated using an acellular tissue engineered vessel (ATEV). At an average of 23.3 months of follow-up, 11 of 12 patients had secondary patency and 7 of 12 had primary patency. One ATEV was removed/abandoned. There were no amputations of ATEV-treated limbs, and no deaths were attributed to the ATEV.
• **Take Home Message:** Treatment using the ATEV resulted in 100% limb salvage and good patency of the ATEV conduit. The ATEV may represent a treatment option for patients suffering the sequelae and complications of vascular procedures of the extremities who lack autologous vein.

Complications and sequelae of vascular surgery, including planned excision of vessels in oncologic procedures, iatrogenic arterial injuries, and steal syndrome after arteriovenous access placement, are increasingly common in modern health care practice.^1^^,^^2^ Although the literature on iatrogenesis is sparse, vascular injury rates have been documented to be increasing over time. The most common mode of iatrogenic injury is percutaneous interventional access, which lead to injury of the femoral artery. Other surgical procedures, including tumor operations and orthopedic procedures, can lead to the need for vascular repair or replacement.^3^

Acellular tissue engineered vessel (ATEV) is a bioengineered, acellular human blood vessel. ATEV was approved by the US Food and Drug Administration in December 2024 for urgent repair in adults as a vascular conduit for extremity arterial injury, when urgent revascularization is needed to avoid imminent limb loss and when autologous vein grafting is not feasible. The vessel is 6 mm in diameter and 42 cm in length, cultured from adult human vascular cells, and rendered acellular at the end of the manufacturing process.^4^ The ATEV is composed primarily of human extracellular matrix proteins found in native vasculature and has suture retention and burst pressures that are similar to those of native arteries.^5^ After implantation, the conduit repopulates with cells from the patient, producing a living blood vessel.^6^, ^7^, ^8^ The ATEV has a shelf life of 18 months under refrigerated conditions and requires no rinsing or preparation steps; thus, it can be rapidly available at the time of clinical need.

The potential advantages of the ATEV in addressing sequelae of vascular surgical procedures may be severalfold. When an arterial injury occurs in the operating room, harvesting saphenous vein can be cumbersome owing to required changes in patient positioning, draping, and the attendant interruption of the index surgical procedure. In addition, unanticipated vein harvest and additional surgical incisions create unintended morbidity for patients.

The ATEV has been evaluated in several indications, including the repair of acute traumatic injuries. Vascular trauma repair in both civilian (CLN-PRO-V005, clinicaltrials.gov NCT03005418) and military (CLN-PRO-V017, clinicaltrials.gov NCT05873959) populations has been studied, and results of these studies have been reported previously.^9^^,^^10^ Prior reported outcomes of the ATEV in treatment of arterial trauma have focused on community-acquired injuries such as gunshot wounds, motor vehicle collisions, and industrial accidents. The goal of this publication was to review the outcomes in the first series of 12 patients wherein ATEV was used to address sequelae or complications of vascular procedures that occurred in the hospital, within the context of the CLN-PRO-V005 clinical trial. The data and outcomes regarding these 12 patients have not been published previously.

## Methods

### Study design

The CLN-PRO-V005 trial (hereafter V005) is a prospective phase 2/3 clinical trial that enrolled 72 patients having vascular injuries at level 1 trauma centers in the United States and Israel from September 2018 to August 2023. The V005 trial received institutional review board approval at all clinical sites before the enrollment of subjects. V005 is a single-arm trial, with no active comparator and no randomization. Patients with either community-acquired injuries of the limbs and torso or experiencing sequelae or complications of vascular surgical procedures in the hospital were consented and enrolled in the trial and received the ATEV for treatment. At the time of enrollment, autologous vein was deemed not feasible for repair in any of the included patients.

**Fig 1:**
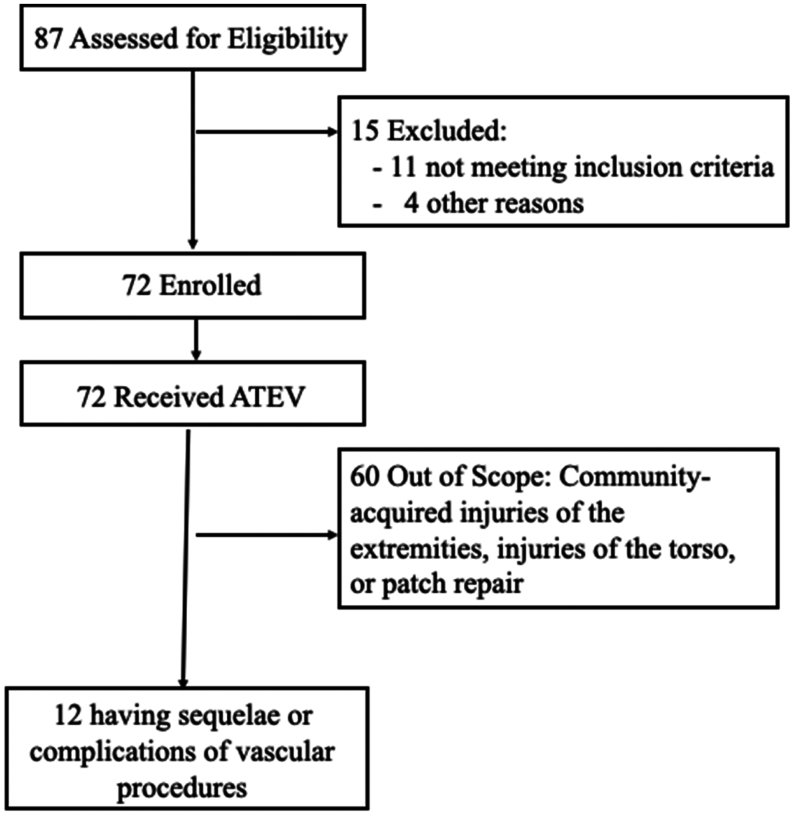
CONSORT diagram for patients treated in the V005 study. *ATEV*, acellular tissue engineered vessel.

Of the 72 patients enrolled in V005, a total of 54 patients were treated for community-acquired extremity vascular injuries. The remaining 18 patients treated in V005 included those with torso injuries, where ATEV was used as a patch (n = 1) and patients treated for sequelae or complications of vascular surgical procedures (n = 12) (Fig 1). This report describes the outcomes in the 12 patients treated with the ATEV in V005 for sequelae or complications of vascular surgical procedures. These patients were enrolled at three separate level 1 trauma centers in the United States. The first patient was treated on March 27, 2019, and the last was treated on April 8, 2022. Of these 12 patients, 5 had iatrogenic injuries that incurred during surgical or percutaneous procedures, 3 underwent planned arterial resection as part of an oncologic procedures, and 4 underwent distal revascularization and interval ligation (DRIL) procedures for ischemia related to prior arteriovenous fistula construction.

The primary efficacy end point for V005 was primary patency of the ATEV at 30 days, and secondary end points included secondary patency, limb salvage, conduit infection, and patient survival, all at 30 days. Safety and efficacy data, including all adverse events and adverse events of special interest relevant to the function of the ATEV, were collected throughout follow-up. Patient follow-up continued until month 36 after ATEV placement, or until patient withdrawal or death, until explantation of the ATEV, or until data cutoff for this report, which was January 15, 2024.

### Statistical analyses

Kaplan-Meier survival analysis was performed to estimate graft patency following the intent-to-treat principle. The Kaplan-Meier analysis of patency and limb salvage was limited to those patients undergoing repair of iatrogenic injuries, and patients receiving the ATEV to replace arteries excised during tumor operations (n = 8 patients total). The four patients who received the ATEV for DRIL procedures were not included in the Kaplan-Meier analysis, but their results are provided descriptively. Patients who experienced intercurrent events, such as death or loss to follow-up unrelated to the study access, were censored at the time of the event for purposes of the Kaplan-Meier analysis, consistent with standard Kaplan-Meier methodology.^11^^,^^12^ Censored events included patients who died or withdrew early with documented graft patency and no evidence of failure.

## Results

### Overview of patients analyzed

Patients were equally split between male and female, with an average age of 57.0 ± 16.9 years (Table I). This group of patients was more female and older than most populations with community-acquired vascular trauma.^13^, ^14^, ^15^ The age range was 28 to 81 years. The majority of patients were White (58%); 33% were Black and 8% Native American/Pacific islander. Levels of comorbidities were high, with 58% of patients having hypertension, 33% having diabetes, 58% having coronary or other heart disease, and 25% having peripheral artery disease.

**Table I:** Patient demographics and implant characteristics

Characteristic	Patients with sequelae and complications of vascular procedures (n = 12)
Demographics	
White	7 (58)
Black/African American	4 (33)
Other: Native American/Pacific islander	1 (8)
Male	6 (50)
Mean age	57.0 ± 16.9
Minimum and maximum age, years	28, 81
Length of ATEV implanted, cm	12.1 ± 9.8

*ATEV,* Acellular tissue engineered vessel.

Values are number (%) or mean ± standard deviation.

The length of conduit used was relatively short: 12.1 ± 9.8 cm. All ATEV conduits were placed as interposition or bypass grafts to reconstruct the arteries in question. No primary repairs and no patch repairs were included in this report.

### Characteristics of vascular complications and sequelae

**Fig 2:**
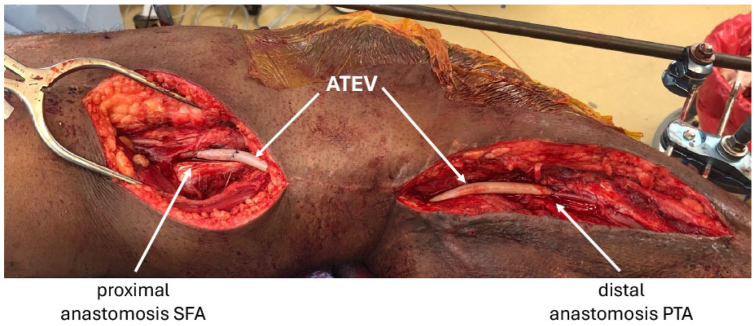
Revascularization using acellular tissue engineered vessel (*ATEV*) after injury to prior saphenous vein graft. A 29-year-old man who had experienced prior popliteal artery injury that had been repaired using saphenous vein suffered injury to the vein graft during an orthopedic procedure. Blood flow to the limb was restored using an ATEV extending from the superficial femoral artery (SFA) to the posterior tibial artery (PTA).

The mechanisms underlying the need for ATEV implantation were as expected for tertiary care medical centers (Table II). Eight implantations occurred in the lower limbs, and four occurred in the upper limbs. Percutaneous vascular interventions to treat peripheral artery or cardiac disease led to four iatrogenic injuries, all in the lower extremities (femoral arteries). Three planned arterial excisions occurred in the setting of tumor resections that were performed in the lower extremities, which necessitated replacement of excised superficial femoral or popliteal artery segments with the ATEV. Four cases of steal syndrome, caused by prior arteriovenous fistula creation for hemodialysis access, were treated using the ATEV. Last, one orthopedic operation led to damage of a preexisting saphenous vein graft in the lower limb, which was replaced using the ATEV (Fig 2).

**Table II:** Sequelae and complications of vascular procedures treated with acellular tissue engineered vessel (*ATEV*) in the V005 trial, by patient

Patient	Age, years	Gender	Mechanism	Affected artery	Length of ATEV implanted, cm
1	29	Male	Orthopedic injury of existing saphenous vein graft that was treating a prior sports injury	Vein graft bypass from SFA to PTA	22
2	81	Male	Cardiac catheterization injury to SFA	SFA	3
3	61	Male	Sarcoma removal involving SFA	SFA	4.5
4	28	Female	Injury to CFA owing to prior thrombectomy procedure	CFA	6
5	62	Male	Excision of mass involving SFA and PA	SFA and PA	8.5
6	69	Female	Cardiac catheterization injury to SFA	SFA	3
7	42	Female	SFA removal during osteosarcoma resection	SFA and PA	15
8	72	Female	BA, setting of arteriovenous fistula and steal syndrome	BA, DRIL procedure	13
9	58	Male	BA, setting of arteriovenous fistula and steal syndrome	BA, DRIL procedure	10
10	54	Male	BA, setting of arteriovenous fistula and steal syndrome	BA, DRIL procedure	11
11	54	Female	BA, setting of arteriovenous fistula and steal syndrome	BA, DRIL procedure	11
12	75	Female	Wire perforation of PA	PA	38

*BA,* Brachial artery; *CFA,* common femoral artery; *DRIL,* distal revascularization and interval ligation; *PA,* popliteal artery; *PTA,* posterior tibial artery; *SFA,* superficial femoral artery.

### Efficacy outcomes for the ATEV

Overall, patients were followed for an average of 23.3 ± 15.9 months. The outcomes for all patients, for the primary end point of 30 days and at the conclusion of follow-up, are shown in Table III. At day 30, nine evaluable patients had primary patency, and one evaluable patient had secondary patency. One of the two remaining patients withdrew consent on day 2, while the other patient died on day 12 of causes unrelated to the ATEV, as adjudicated by an independent committee. At day 30, there were no instances of ATEV conduit infection and no amputations of the treated limbs.

**Table III:** Acellular tissue engineered vessel (*ATEV*) arterial repair outcomes by patient

Patient	Patency at day 30	Follow-up duration	Patency at study completion, data cutoff, death or withdrawal	Conduit infected?	Amputation?	Range of conduit diameters on duplex ultrasound examination, mm	Censoring event
1	Secondary	36.0 months	Secondary	No	No	4.6-5.5	36-month cutoff
2	Primary	36.0 months	Primary	No	No	4.2-6.0	36-month cutoff
3	Primary	36.8 months	Primary	No	No	5.4-6.0	Death, unrelated to ATEV
4	--	1 day	Primary	No	No	--	Withdrew consent
5^a^	Primary	40 days	Failed (Explanted)	Possibly^a^	No	5.8	ATEV removed
6	Primary	33.6 months	Primary	No	No	3.7-7.6	Data cutoff^b^
7	Primary	20.3 months	Primary	No	No	5.0-6.0	Data cutoff
8	--	12 days	Primary	No	No	--	Death, unrelated to ATEV
9	Primary	32.8 months	Primary assisted	No	No	5.3-7.2	Death, unrelated to ATEV
10	Primary	35.9 months	Primary	No	No	5.5-6.7	36-month cutoff
11	Primary	9.4 months	Primary assisted	No	No	6.1	Death, unrelated to ATEV
12	Primary	36.0 months	Primary assisted	No	No	5.7-6.8	36-month cutoff
							
ALL	--	23.3 ± 15.9 months	--	No	No	3.7-7.6	--

a Details of patient clinical sequence can be found in Appendix (online only), patient narrative 5.

b Data cutoff January 15, 2024.

**Fig 3:**
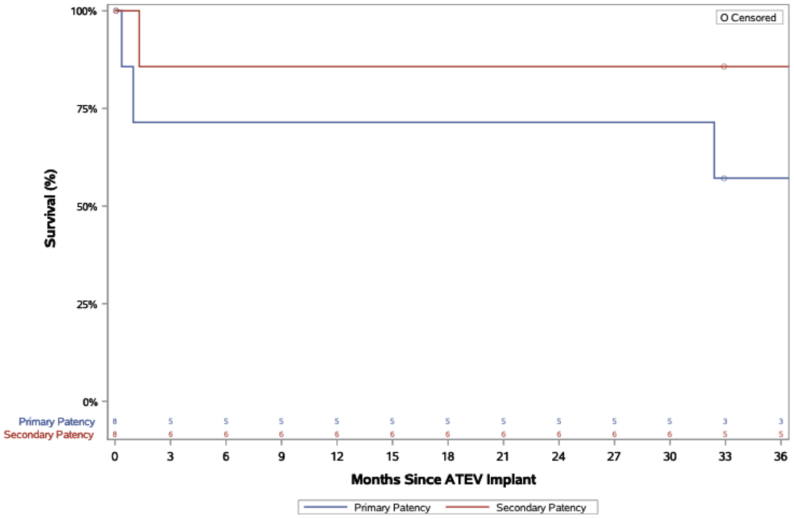
Kaplan-Meier curves of primary and secondary patency for acellular tissue engineered vessel (*ATEV*) in eight patients. ATEV primary and secondary patency are shown for the 36-month duration of follow-up in eight patients treated for sequelae or complications of vascular procedures. Patients are censored for intercurrent events such as death not attributable to the ATEV or loss to follow-up. The numbers shown below the Kaplan-Meier curve at each timepoint represent the number of patients remaining at risk for the event at that interval.

Efficacy outcomes are divided into those for the 8 patients who received the ATEV for a non-DRIL procedure, that is, iatrogenic repair and replacement of excised arteries during oncologic surgery, and the four patients who underwent a DRIL using the ATEV. As of April 10, 2025, among the eight patients receiving ATEV for non-DRIL procedures, three lost primary patency and one lost secondary patency. Kaplan-Meier curves showing primary and secondary patency, with censoring for patients who died or who were lost to follow-up in the setting of a patent conduit,^16^ are shown in Fig 3.

In one patient, the ATEV was removed at day 40 owing to mid-graft bleeding after repeated reinterventions on the conduit. This patient (Appendix, online only, patient 5) required vascular reconstruction after a large repeat tumor resection and then developed an infected perivascular hematoma. After surgical evacuation of the hematoma on day 39, ATEV disruption in the mid portion of the graft and bleeding was identified and led to resection of the ATEV on day 40. This case was counted as a conduit failure and as a loss of secondary patency. Although no bacteria were visible in the wall of the resected ATEV by histological analysis, the infected perivascular hematoma showed abundant bacteria in the perigraft space.

Throughout follow-up, there were no amputations of the treated limbs, and no confirmed conduit infections were reported, although perigraft infection contributing to ATEV disruption could not be excluded definitively for patient 5.

Of the four patients who received ATEV for DRIL procedures (patients 8, 9, 10, and 11) (Table III), one patient died at day 12 owing to unrelated reasons. The ATEV was patent and functioning at the time of the death. The other three DRIL patients retained primary patency at day 30 and retained primary or primary-assisted patency over the duration of their follow-up. One patient was followed for 9.4 months until death, and one patient was followed for 32.8 months until death; both deaths were due to reasons unrelated to the ATEV. The fourth DRIL patient completed 3 years of follow-up.

### Safety outcomes for the ATEV

Evaluation of the safety of the ATEV indicated no safety signals attributable to ATEV mechanical weakness, product contamination, or immune rejection. Overall, adverse events and serious adverse events were frequent in V005 and were consistent with patients suffering from sequelae and complications of vascular procedures, in the setting of other diseases and comorbidities (Table IV).

**Table IV:** Adverse events (*AEs*) and adverse events of special interest (*AESIs*)

Safety events during entirety of follow-up	Treated patients (n = 12)
AEs	
Mild	11 (92)
Moderate	10 (83)
Severe	8 (67)
Life-threatening	0
Deaths	4 (33)
AESIs	
ATEV conduit infection	0
ATEV rupture	1^a^
ATEV occlusion/thrombosis	2 (17)
ATEV pseudoaneurysm	1 (8)
ATEV aneurysm	0

*ATEV,* Acellular tissue engineered vessel.

Values are number (%).

a ATEV mid-graft disruption and bleeding with peri-graft infection (Appendix, online only, patient narrative 5).

Adverse events of special interest, including thrombosis, rupture, aneurysm, and pseudoaneurysm, occurred at acceptable rates that were consistent with reports of other vascular conduits in the setting of vascular trauma, including autologous vein and synthetic grafts.^17^, ^18^, ^19^ On duplex ultrasound examination, the measured diameter of the 6 mm ATEV was typically in the range of 5 to 7 mm throughout follow-up (Table III), with a small number of measurements outside of this range. There were no reports of aneurysm in any patient.

There were two reports of thrombosis during the follow-up period that were corrected percutaneously with restoration of patency. There was one case of ATEV midgraft disruption and bleeding that occurred within days of a repair of an anastomotic pseudoaneurysm with stent placement, in the setting of an adjacent infected hematoma (Appendix, online only, patient 5). In this case, the ATEV was excised and replaced with autologous vein on day 40.

There were 4 deaths out of 12 treated patients in this cohort during the follow-up period. One patient died on day 12 of influenza A respiratory infection in the setting of a patent ATEV, and a second patient died at month 9.4 of COVID-related pneumonia. One patient died at 34.7 months of unknown causes in the setting of end-stage renal failure. The last patient died at 36.8 months in the setting of multiple, previously diagnosed, metastatic primary carcinomas and metastatic disease in the lung. None of these deaths was deemed related to the ATEV, as determined by surgical investigators and by adjudication (for the patient who died on day 12). Brief narrative summaries of the injuries and outcomes for each patient are included in the Appendix (online only).

## Discussion

Increased use of vascular and percutaneous procedures has led to increases in concomitant sequelae and iatrogenic injuries. A review of the national rates of hospital-acquired vascular trauma in Sweden showed a 115% increase from 1987 to 2005.^1^ Similarly, rates of physician-generated vascular injuries more than doubled at a US medical center from 1994 to 2002.^2^ The increased use of percutaneous and minimally invasive procedures, while decreasing overall patient morbidity and benefiting recovery time, has been associated with increasing rates of inadvertent injuries to the native vasculature.

In the cohort reported here, comorbidities were common. High rates of cardiovascular disease in these patients meant that autologous vein may not have been available for repair when an event occurred. Patients with preexisting coronary or peripheral vascular disease may have undergone prior vein harvest or may benefit from retaining their saphenous vein for use in future revascularization procedures.

Hospitalized patients are typically older, sicker, and have more comorbidities than patients with community-acquired vascular trauma. From the sample in this report, gender was balanced, which is in contrast with noniatrogenic trauma populations, which are predominantly male.^20^ Mortality in this group was as expected for a hospitalized population undergoing vascular procedures: 30-day mortality was 8.3%, which is similar to the 30-day mortality of approximately 7% that has been reported for iatrogenic vascular injuries.^2^ In the longer term, overall mortality was 33% over an average of 23.2 months of follow-up, which is higher than long-term mortality rates reported for noniatrogenic injuries, but likely reflects the overall burden of comorbidities in these patients.

In this series of prospectively studied patients, there was 100% limb salvage and no deaths attributed to the ATEV. There were no reports of aneurysm, and the diameters of the ATEV as measured by duplex ultrasound examination were close to the original 6-mm diameter of the conduit. There was one ATEV failure that occurred around day 40 in the setting of wound infection and an infected perivascular hematoma. Histological examination of the ATEV defect, determined to be the site of bleeding, was consistent with an iatrogenic cause. Bacteria were not observed in the wall of the conduit, although on Gram stain bacteria were abundant outside of vessel in the perivascular space (Appendix, online only, patient 5).

The ATEV was used to repair or replace the common femoral, superficial femoral, popliteal, posterior tibial, and brachial arteries. Over the follow-up period, 1 of the 12 ATEV conduits failed and was removed; the others retained patency. These data imply that ATEV may be an effective and durable conduit for vascular repair in hospitalized patients suffering vascular sequelae or complications during hospital care.

As with any early clinical experience, there are several limitations to this report. The patients described herein comprise a heterogeneous group, with a wide range of mechanisms and comorbidities. Literature reports of hospital-acquired vascular injury repairs, including iatrogenic repairs are too few, and too sporadic, to provide any reliable benchmarks. Therefore, to truly understand ATEV function in an array of hospital-acquired injuries, more long-term study is needed. The ATEV conduit, when used for revascularization of these sequelae and complications of vascular procedures, may offer important benefits for patients and for the health care system.

## Conclusions

There have been very few novel vascular conduits introduced into clinical practice in the last several decades, and none have been proven superior to polytetrafluoroethylene. The ATEV is a novel, bioengineered conduit that has dual benefits of biological composition, while being immediately available and off the shelf. Moreover, ATEV appears to demonstrate a favorable response in the setting of infection.^6^ The results in this report provide evidence that, in patients requiring vascular conduit owing to planned arterial excisions, iatrogenic injuries, or DRIL procedures, and who lack an autologous vein, the ATEV may provide a valuable therapeutic alternative. Further study is needed to confirm these results in larger patient populations.

## Funding

These clinical studies and systematic literature review were supported by Humacyte Global, Inc. The sponsor participated in the design and conduct of the studies collection, management, analysis, and interpretation of the data; preparation, review and submission of the manuscript.

## Disclosures

E.E.M., R.K., Y.W.L., J.M., and J.T.S. received research support from Humacyte, the sponsor of the study reported in this publication. L.E.N. is the founder and CEO of Humacyte, member of the Board, receives a salary, owns stock, and multiple patents either owned or licensed by Humacyte. S.P. is the CMO of Humacyte, receives a salary, and owns stock.
