# Supplementary material for: Bioengineered human blood vessels to treat hospital-acquired vascular complications
Source: J Vasc Surg Cases Innov Tech. 2025 Sep 8;11(6):101976. doi: 10.1016/j.jvscit.2025.101976 (PMC12547166; doi:10.1016/j.jvscit.2025.101976)
Supplement: Supplementary Material [file mmc1.docx]

**Appendix: Patient Narratives**

Patient #1:

In November 2018 a 29 yo otherwise healthy male who sustained a sports injury of left posterior knee dislocation with injury to popliteal artery, which was repaired using saphenous vein. Four months later, the patient was undergoing an orthopedic procedure for ligamentous reconstruction when the saphenous vein graft was damaged, requiring ligation of the conduit. Subsequently, the left lower leg was pulseless, and the patient received an ATEV to repair the damaged saphenous vein graft, from the superficial femoral artery to the posterior tibial artery (Figure 2). On day 11, a balloon thrombectomy was performed on the posterior tibial artery associated with focal dissection. The patient was discharged on day 16. Follow-up duplex ultrasound showed an occluded ATEV conduit at month 3, but a patent conduit at month 9, with no intervening intervention, and the distal leg/foot remained well perfused. Patient completed the study at 36 months and maintained secondary patency and limb salvage.

Patient #2:

Patient is an 81 yo male with iatrogenic injury to the common femoral artery following a cardiac catheterization in August 2019. Patient had a history of heart disease and a prior vein harvest for CABG, and so the ATEV was utilized to repair the injury, extending from the right common femoral artery to the proximal superficial femoral artery. The patient suffered no adverse events related to the ATEV and completed the study at 36 months, at which time primary patency of the conduit was documented by Duplex ultrasound.

Patient #3:

Patient is a 61 yo male with history of multiple primary carcinomas, who underwent surgery to remove a sarcoma in the left thigh in October 2019. The tumor resection required excision of a portion of the left superficial femoral artery, which was repaired using an ATEV interposition graft. There were no adverse events related to the ATEV throughout follow-up until month 27.6, with primary patency and limb salvage maintained, and with no evidence of conduit infection. Study participation ended at month 36.8 due to death of the patient, determined to be unrelated to the ATEV.

Patient #4:

Patient is a 28 yo female with history of carotid dissection and stroke, who developed a right common femoral artery dissection at the access site for a prior percutaneous procedure in May 2020. In July 2020, the patient presented with pallor of the right lower extremity and was taken to the operating room for femoral artery repair, at which time the saphenous vein was deemed not suitable. The patient’s spouse gave verbal consent intra-operatively for use of the ATEV for repair of the common femoral artery, which was performed without complication. On day 2, when the patient recovered from anesthesia, she withdrew consent and so participation in the trial was terminated at that time. At time of withdrawal of consent, the ATEV had primary patency.

Patient #5:

Patient is a 62 yo male with history of widely metastatic renal cell carcinoma, who underwent wide resection of a right thigh mass in October 2020. Tumor resection included excision of vasculature in the thigh, and the artery was replaced by an ATEV extending from the superficial femoral artery to the popliteal artery. At day 30, the patient developed a pseudoaneurysm of the distal anastomosis with contained rupture. This pseudoaneurysm was successfully resolved by percutaneous placement of a covered stent to exclude the pseudoaneurysm, and the patient was discharged without requiring ATEV explantation, additional intervention, or vein grafting. On day 37, the patient developed fever and CT scan revealed a likely abscess in the right thigh abutting the ATEV, at which time the patient was started in IV antibiotics. The infected right thigh hematoma was surgically evacuated on day 39, and cultures were positive for *Enterococcus* and *E. coli*. Both the stented distal anastomosis and the proximal ATEV anastomosis were intact and free of bleeding. However, on day 40, sudden bleeding from the right thigh drain necessitated a return to the operating room, at which time bleeding from an area of dehiscence in the midportion of the ATEV graft was identified. The ATEV was removed and replaced with an interposition vein graft. Subsequent histological examination of the explanted ATEV revealed a linear, full-thickness wall defect with sharply demarcated edges, consistent with mechanical trauma - likely due to a sharp surgical instrument. The location and morphology of the defect suggest an iatrogenic cause, potentially incurred during prior intervention. Inflammatory findings were consistent with a recent injury, lacking features of chronicity. Bacteria were not observed in the wall of the ATEV, but gram-positive cocci were present in tissue outside of the ATEV. The patient remained limb-intact but was discontinued from the study on day 40 due to loss of secondary patency following ATEV removal.

Patient #6:

Patient is a 69 yo female with history of atrial fibrillation who underwent percutaneous nodal ablation complicated by injury to the right common femoral artery, in January 2021. The arterial injury was repaired with a segment of ATEV. The patient experienced no adverse events related to the ATEV, and maintained primary patency and limb salvage throughout follow-up.

Patient #7:

Patient is a 42 yo female with history of parosteal osteosarcoma of the left distal femur that underwent initial resection in 2017. In April 2022, the patient returned to the OR for re-excision of the tumor mass, which necessitated excision of the left femoral artery and vein. A segment of ATEV was utilized for repair from the superficial femoral to the above-knee popliteal artery, without complication. No adverse events were attributed to the ATEV, and primary patency and limb salvage were documented through Month 20. Follow-up is ongoing.

Patient #8:

Patient was a 72yo women with end-stage renal disease on hemodialysis, who developed steal syndrome following brachiocephalic arteriovenous fistula creation. To treat the steal symptoms, the ATEV was placed as a brachial artery interposition graft in March 2019 without complications. On day 8, the patient developed shortness of breath, weakness, and chest pain, which led to a diagnosis of Influenza A. Despite supportive care, the patient deteriorated and died on day 12 due to respiratory failure and acute encephalopathy. Since this patient died within 30 days of ATEV implantation, the details of the case were sent to an independent adjudication committee and the death was adjudicated as not related to the ATEV, which had maintained primary patency.

Patient #9:

Patient is a 58 yo male with multiple medical problems including end-stage kidney disease on hemodialysis. The patient underwent arteriovenous fistula creation in 2018 which was complicated by steal syndrome. In March 2019, the patient underwent a Distal Revascularization and Interval ligation (DRIL) procedure using the ATEV to treat the symptoms of steal. At month 17, there was an angioplasty performed to treat localized stenosis of the ATEV, and primary patency was lost at that time. The patient continued in follow-up until Month 33 with no further interventions on the ATEV. Subsequent to the Month 33 visit, the patient died of unknown causes that were unrelated to the ATEV, and assigned patency status was primary assisted patency.

Patient #10:

Patient is a 54 yo male who suffered steal syndrome following creation of a brachiocephalic arteriovenous fistula for dialysis access six months earlier. Patient underwent a DRIL procedure in April 2019 to correct the symptoms of arterial steal in the left hand. No adverse events were attributed to the ATEV, and which retained primary patency through the 36-month follow-up period.

Patient #11:

Patient is a 54 yo female with end-stage renal disease who developed vascular steal syndrome following brachio-basilic arteriovenous fistula creation, due to stenosis of the brachial artery. The patient received an ATEV in August 2019 to bypass the stenosed brachial artery. At month 5.5, fistulogram showed stenosis of the AV anastomosis, not involving the ATEV, which was treated with balloon angioplasty. At this point the patient had primary assisted patency of the ATEV. At month 9, the patient was diagnosed with COVID-19 pneumonia, and died several weeks thereafter due to hypoxic respiratory failure.

Patient #12:

Patient is a 75 yo female with multiple medical problems who suffered a wire perforation of a popliteal artery during a percutaneous revascularization procedure. Due to time limitations making vein harvest not possible, and the poor quality and size of the veins, the patient received an ATEV implant as a femoro-popliteal bypass in the right leg in October 2019. There were no adverse events attributable to the ATEV until month 12.8, when angiography showed mild narrowing of the proximal and distal anastomoses. Treatment was not undertaken until month 32.4, when ATEV stenosis was treated with angioplasty. The ATEV retained primary assisted patency at the Study Completion Month 36 time point but was abandoned after 36 months at month 37.
